# Cerebral blood flow and cognition after 3 months tadalafil treatment in small vessel disease (ETLAS-2): study protocol for a randomized controlled trial

**DOI:** 10.1186/s13063-024-08402-4

**Published:** 2024-08-29

**Authors:** Joakim Ölmestig, Kristian Nygaard Mortensen, Birgitte Fagerlund, Nadia Naveed, Mette Maria Nordling, Hanne Christensen, Helle Klingenberg Iversen, Mai Bang Poulsen, Hartwig Roman Siebner, Christina Kruuse

**Affiliations:** 1 Neurovascular Research Unit, Department of Neurology, Copenhagen University Hospital—Herlev and Gentofte, Copenhagen, Denmark; 2 Department of Clinical Medicine, University of Copenhagen, Copenhagen, Denmark; 3 Danish Research Centre for Magnetic Resonance, Centre for Functional and Diagnostic Imaging and Research, Copenhagen University Hospital—Amager and Hvidovre, Copenhagen, Denmark; 4 Child and Adolescent Mental Health Center, Copenhagen University Hospital, Mental Health Services CPH, Copenhagen, Denmark; 5 Department of Psychology, University of Copenhagen, Copenhagen, Denmark; 6 Department of Radiology, Copenhagen University Hospital—Herlev and Gentofte, Copenhagen, Denmark; 7 Department of Neurology, Copenhagen University Hospital—Bispebjerg and Frederiksberg, Copenhagen, Denmark; 8 Department of Neurology, Copenhagen University Hospital—Rigshospitalet, Copenhagen, Denmark; 9 Department of Neurology, Copenhagen University Hospital—North Zealand, Copenhagen, Denmark; 10 Department of Brain and Spinal Cord Injury, Neuroscience Centre, Copenhagen University Hospital—Rigshospitalet, Copenhagen, Denmark

**Keywords:** Stroke, Dementia, ASL-MRI, BOLD-dependent MRI, Blood–brain barrier, Cognitive testing

## Abstract

**Background:**

Targeted treatment is highly warranted for cerebral small vessel disease, a causal factor of one in four strokes and a major contributor to vascular dementia. Patients with cerebral small vessel disease have impaired cerebral blood flow and vessel reactivity. Tadalafil is a specific phosphodiesterase 5 inhibitor shown to improve vascular reactivity in the brain.

**Methods:**

The ETLAS-2 trial is a phase 2 double-blind, randomized placebo-controlled, parallel trial with the feasibility of tadalafil as the primary outcome. The trial aims to include 100 patients with small vessel occlusion stroke or transitory ischemic attacks and signs of cerebral small vessel disease more than 6 months before administration of study medication. Patients are treated for 3 months with tadalafil 20 mg or placebo daily and undergo magnetic resonance imaging (MRI) to evaluate changes in small vessel disease according to the STandards for ReportIng Vascular changes on nEuroimaging (STRIVE) criteria as well as cerebral blood flow, cerebrovascular reactivity, and neurovascular coupling in a functional MRI sub-study. The investigation includes comprehensive cognitive testing using paper–pencil tests and Cambridge Neuropsychological Test Automated Battery (CANTAB) tests in a cognitive sub-study.

**Discussion:**

The ETLAS-2 trial tests the feasibility of long-term treatment with tadalafil and explores vascular and cognitive effects in cerebral small vessel disease in trial sub-studies. The study aims to propose a new treatment target and improve the understanding of small vessel disease. Currently, 64 patients have been included and the trial is estimated to be completed in the year 2024.

**Trial registration:**

Clinicaltrials.gov, NCT05173896. Registered on 30 December 2021.

**Supplementary Information:**

The online version contains supplementary material available at 10.1186/s13063-024-08402-4.

## Administrative information

Note: the numbers in curly brackets in this protocol refer to SPIRIT checklist item numbers. The order of the items has been modified to group similar items (see http://www.equator-network.org/reporting-guidelines/spirit-2013-statement-defining-standard-protocol-items-for-clinical-trials/).

**Table:** 

Title {1}	Cerebral blood flow and cognition after three months tadalafil treatment in small vessel disease (ETLAS-2): study protocol for a randomized controlled trial.
Trial registration {2a and 2b}.	Clinicaltrials.gov: NCT05173896. Registered on 30th of December 2021. Clinicaltrialsregister.eu: EudraCT number: 2020-002329-27. Registered on 10th of December 2020.
Protocol version {3}	Protocol version: 6, Date: 17th of May 2022.
Funding {4}	This study is supported by Novo Nordisk Foundation – Investigator Initiated Clinical Trials (NNF20OC0063912), Frimodt-Heineke Foundation (no grant number), Foundation for Research in Neurology (no grant number), and Herlev and Gentofte Hospitals Research Fund (no grant number).
Author details {5a}	Joakim Ölmestig^1,2,3^, Kristian Nygaard Mortensen^3^, Birgitte Fagerlund^4,5^, Nadia Naveed^6^, Mette Maria Nordling^6^, Hanne Christensen^2,7^, Helle Klingenberg Iversen^2,8^, Mai Bang Poulsen^9^, Hartwig Roman Siebner^2,3,7^, Christina Kruuse^1,2,10*^ ^1^ Neurovascular Research Unit, Department of Neurology, Copenhagen University Hospital – Herlev and Gentofte, Copenhagen, Denmark ^2^ Department of Clinical Medicine, University of Copenhagen, Copenhagen, Denmark ^3^ Danish Research Centre for Magnetic Resonance, Centre for Functional and Diagnostic Imaging and Research, Copenhagen University Hospital – Amager and Hvidovre, Copenhagen, Denmark ^4^ Child and Adolescent Mental Health Center, Copenhagen University Hospital, Mental Health Services CPH, Copenhagen, Denmark ^5^ Department of Psychology, University of Copenhagen, Copenhagen, Denmark ^6^ Department of Radiology, Copenhagen University Hospital – Herlev and Gentofte, Copenhagen, Denmark ^7^ Department of Neurology, Copenhagen University Hospital – Bispebjerg and Frederiksberg, Copenhagen, Denmark ^8^ Department of Neurology, Copenhagen University Hospital – Rigshospitalet, Copenhagen, Denmark ^9^ Department of Neurology, Copenhagen University Hospital – North Zealand, Copenhagen, Denmark ^10^ Department of Brain and Spinal Cord Injury, Neuroscience Centre, Copenhagen University Hospital – Rigshospitalet, Copenhagen, Denmark
Name and contact information for the trial sponsor {5b}	Department of Brain and Spinal Cord Injury, Neuroscience Centre, Copenhagen University Hospital – Rigshospitalet, Valdemar Hansens Vej 13, 2600 Glostrup, Denmark & Neurovascular Research Unit, Department of Neurology, Copenhagen University Hospital – Herlev and Gentofte, Borgmester Ib Juuls Vej 1, 2730 Herlev, Denmark. Sponsor delegate person: Christina Kruuse Email: ckruuse@dadlnet.dk
Role of sponsor {5c}	This trial is an investigator initiated clinical trial. The sponsor delegate person is the initiator of the study, involved in study design, MRI design, data analysis, and writing of reports. The sponsor can decide on submitting reports for publication and has authority over data and manuscript submission. The funders are not involved in the study design and in conducting the trial. Funders have no right to data from this trial.

## Introduction

### Background and rationale {6a}

In this trial, we explore a treatment targeting cerebrovascular regulation in cerebral small vessel disease (CSVD). CSVD is the underlying cause of 25% of ischemic cerebral strokes and is involved in most hemorrhagic strokes [1, 2]. Further, CSVD is the primary cause of vascular dementia, the most frequent dementia aside from Alzheimer’s disease [2–4]. Finding a treatment for CSVD will significantly impact the burden of stroke and dementia.

CSVD is defined by changes in the small vessel structure and function with reduced cerebral blood flow (CBF) and cerebrovascular reactivity (CVR) [5–9]. Its pathophysiology involves endothelial dysfunction which is associated with impaired endothelial nitric oxide (NO) production and reduced cyclic guanosine monophosphate (cGMP) within the smooth muscle cells of the vascular wall [10–12]. The NO-cGMP signaling is key to regulating blood flow in the body and a disruption of this signaling is likely to be involved in CSVD pathology [13–15]. Phosphodiesterase 5 (PDE-5) inhibitors, such as tadalafil, are pharmacological agents that reduce the breakdown of cGMP and therefore augment the NO-cGMP signaling [16]. PDE-5 inhibitors are currently used to treat erectile dysfunction and pulmonary hypertension [16]. Since the PDE-5 enzyme is also expressed in human brain and brain vasculature, PDE-5 inhibitors also act on the cerebral vasculature [17–19].

Improving CBF and CVR with tadalafil could be an attractive mechanism for slowing or reverting the effects of CSVD and reducing the risk of recurrent stroke and dementia. We have previously shown in a pilot study that a single dose of tadalafil 20 mg vs. placebo increased blood oxygen saturation in the cerebral cortex, using a bedside method to estimate regional CBF [20]. Tadalafil and other PDE-5 inhibitors are well-tested, and their side-effect profile is well-known and often tolerated [21–23].

## Objectives {7}

The purpose of this study is to test the feasibility of daily treatment with tadalafil in CSVD patients and explore the effect on CBF and CVR with magnetic resonance imaging (MRI) and changes in cognition. The main hypothesis is that daily treatment with oral tadalafil (20 mg) is feasible and at least 90% of patients in both treatment arms will reach the target dose. Target dose is defined as the intake of study drug in at least 90% of the trial period. The secondary outcomes on CBF, CVR, and cognition are hypothesis generating in exploratory sub-studies to identify the effect on vascular reactivity and cognition. The overall hypothesis is that tadalafil can be a disease-modifying treatment for CSVD.

## Trial design {8}

The ETLAS-2 trial is an investigator-initiated, randomized, placebo-controlled, double-blind, parallel-arm, phase 2 clinical trial in the Capital Region of Denmark. The allocation ratio is 1:1 between tadalafil and placebo. The framework applied is exploratory.

## Methods: participants, interventions, and outcomes

### Study setting {9}

The trial is conducted at the Department of Neurology, Copenhagen University Hospital—Herlev and Gentofte (HGH) in collaboration with Danish Research Centre for Magnetic Resonance (DRCMR), Copenhagen University Hospital—Amager and Hvidovre (AHH), Department of Neurology, Copenhagen University Hospital—Rigshospitalet (RH), Department of Neurology, Copenhagen University Hospital—Bispebjerg and Frederiksberg (BFH), and Department of Neurology, Copenhagen University Hospital—North Zealand (NZH). Patients that have signs of CSVD and late-phase small vessel occlusion stroke/transitory ischemic attack (TIA) (within the previous 5 years) can be included with the start of intervention earliest 6 months post-stroke. The cutoff of 6 months has been chosen because PDE-5 inhibitors are currently contraindicated in the acute phase of stroke based on case reports of cerebral hemorrhage and lack of experience. The aim of this trial is not to treat the acute ischemia but to study the underlying vascular pathophysiology of CSVD Tables 1 and 2.

### Eligibility criteria {10}

**Table 1:** Inclusion criteria

1	MRI/computed tomography (CT) evidence of small vessel occlusion stroke(s)/lacunar stroke(s) (involving ≤ 2 cm in the acute phase and ≤ 1.5 cm in the late phase) and/or white matter hyperintensities (≥ grade 2 on Fazekas’s scale)
2	Clinical evidence of CSVD: (a) small vessel occlusion stroke (lacunar stroke) syndrome with symptoms lasting > 24 h, occurring < 5 years ago OR (b) transient ischemic attack (TIA) with symptoms lasting < 24 h AND an initial MRI diffusion-weighted imaging (DWI) showing a small vessel occlusion stroke, occurring < 5 years ago OR (c) TIA with symptoms lasting < 24 h AND no acute MRI-DWI lesion but MRI/CT evidence of CSVD with old small vessel occlusion stroke(s) and/or WMH (≥ grade 2 on Fazekas’s scale), occurring < 5 years ago
3	Age ≥ 50 years

**Table 2:** Exclusion criteria

1	Known diagnosis of dementia or under investigation for dementia
2	Pregnancy or nursing
3	Women of childbearing age not taking contraception
4	Known cortical infarction (> 1.5 cm maximum diameter)
5	Known carotid artery stenosis ≥ 50% with Doppler ultrasound, CT angiography, or MRI angiography diagnosed within the last 5 years
6	Known carotid or vertebral dissection as a cause of stroke
7	Stroke after carotid or heart surgery
8	Known hypercoagulable disease
9	Systolic BP < 90 and/or diastolic BP < 50
10	Known severe renal impairment (eGFR < 30 ml/min)
11	Known severe hepatic impairment (Child–Pugh > B)
12	History of non-arthritic anterior ischemic optic neuropathy
13	Concomitant use of PDE-5 inhibitors during trial period
14	Patients receiving nicorandil and nitrates, e.g., isosorbide mononitrate, isosorbide dinitrate, and glyceryl trinitrate
15	History of acute myocardial infarction in the last three months before trial intervention
16	Body weight > 130 kg
17	Known cardiac failure (NYHA ≥ II)
18	Known persistent or paroxysmal atrial fibrillation/flutter
19	History of “sick sinus syndrome” or other supraventricular cardiac conduction conditions such as sinoatrial or atrioventricular block (second or third degree)
20	Other known cardiogenic cause of stroke
21	Contraindication to CO_2_ challenge, e.g., severe respiratory disease
22	MRI not tolerated or contraindicated
23	Known monogenic causes of stroke, i.e., CADASIL
24	Unable to provide informed consent
25	The participant does not wish to be informed about the results from the MRI

#### Who will take informed consent? {26a}

Investigators in the trial obtain oral and written informed consent from trial participants after an information visit is done. The information visit is carried out in an uninterrupted room. During the information visit, the trial is explained in detail regarding background, methods, potential side-effects, and what the participants can gain from participating in the trial.

#### Additional consent provisions for collection and use of participant data and biological specimens {26b}

We collect blood samples for a biobank for future research as a separate voluntary study. A separate informed consent is obtained from trial participants who agree to participate.

## Interventions

### Explanation for the choice of comparators {6b}

The trial is designed as a placebo-controlled study with a 1:1 randomization rate between tadalafil and placebo. As we test the feasibility of daily tadalafil treatment in patients with cerebral small vessel disease and its effect on cerebrovascular and cognitive outcomes, placebo treatment was chosen as a comparator.

### Intervention description {11a}

The intervention is a daily intake of tadalafil 20 mg or placebo together with guideline medication for approximately 3 months. Tadalafil or placebo (lactose monohydrate and potato starch) are included in 96 opaque capsules for each participant to ensure blinding. Participants are instructed to take study medication in the morning. The dose of 20 mg tadalafil was based on our previous study [20], a collaborative study conducted at St George’s University of London, UK [24], and that 20 mg is common in other conditions, such as pulmonary hypertension and erectile dysfunction.

### Criteria for discontinuing or modifying allocated interventions {11b}

Participants who repeatedly fail to comply with study agreements can be discontinued. Discontinuation will also occur if the first MRI cannot be conducted as this is considered an important outcome of the trial. In case of serious adverse events due to study medication, the intervention will be stopped. Participants who request to stop study medication, e.g., due to side effects, will be allowed to do so without being discontinued. If study medication is stopped, the participants are encouraged to continue the study without study medication as the feasibility of the treatment is the main outcome. The reason for terminating the study medication is noted.

### Strategies to improve adherence to interventions {11c}

Participants are contacted by a phone call every week for the first month after the study medication has been initiated to promote motivation for the study and to assess side effects and adverse events.

### Relevant concomitant care permitted or prohibited during the trial {11d}

Apart from nitrates (e.g., isosorbide mononitrate, isosorbide dinitrate, and glyceryl trinitrate) and PDE-5 inhibitors, any treatment or medication is permitted. Nitrates are prohibited due to their interaction with PDE-5 inhibitors. Nitrates and concomitant use of PDE-5 inhibitors during the trial period are listed as exclusion criteria.

### Provisions for post-trial care {30}

Damages to participants caused by the study are covered, as described in the Danish Law.

### Outcomes {12}

Outcomes will be analyzed in a main study and the following predefined sub-studies: functional MRI exploratory sub-study, cognitive exploratory sub-study, and biomarker exploratory sub-study. All outcomes in the main study and sub-studies are assessed twice, initially at the baseline visit before study medication is initiated and then at the 3-month follow-up visit at the end of the trial period comparing changes from baseline to follow-up.

#### Main study

##### Primary outcome: feasibility

The proportion of participants achieved full target dose of tadalafil/placebo by the end of the trial period. We allow a 10% failure of tablet intake in the trial period which still counts as full completion of medication. Assessment is done by tablet count and a questionnaire.

##### Secondary outcome: MRI

Cerebral MRI is performed on a 3 T Philips Achieva scanner (Philips, The Netherlands). The following MRI sequences are used for anatomical evaluation: T1w, T2w, fluid-attenuated inversion recovery (FLAIR), diffusion-weighted imaging (DWI), susceptibility-weighted imaging (SWI), and time of flight (TOF). MRIs will be assessed by two independent neuroradiologists according to the STRIVE guidelines from 2013, including white matter hyperintensity (WMH), lacunes, microbleeds, enlarged perivascular space, atrophy, and recent infarcts [25]. We also evaluate the presence of cortical superficial siderosis which is included in the recent STRIVE guidelines from 2023 [26]. WMH volume is assessed using an automatic solution with manual correction based on FLAIR and T1w images [27].

##### Secondary outcome: cognition

Informant Questionnaire on Cognitive Decline in the Elderly (IQCOED) and Montreal Cognitive Assessment (MoCA) are used to assess cognitive function in the main study.

##### Secondary outcome: depression, fatigue, and mental well-being questionnaires

The degree of depression is assessed using Becks Depression Inventory II (BDI-II). Fatigue Severity Scale (FSS) is used to examine fatigue, and WHO-5 Well-Being Index to assess mental well-being.

##### Secondary outcome: safety and adverse events

Adverse events are registered according to good clinical practice from the day of inclusion until the safety follow-up visit is done.

##### Secondary outcome: blood pressure, heart rate, and electrocardiogram

Blood pressure, heart rate, and electrocardiogram are performed.

##### Secondary outcomes: blood samples

Routine blood samples are performed (hemoglobin, hematocrit, leukocytes, platelets, liver-, kidney-, and metabolic function, and high sensitivity C-reactive protein). To control for intake of study medication, plasma levels of tadalafil will be analyzed after the trial is completed.

##### Secondary outcomes: registered-based follow-up

Register-based outcome assessment will be done with a composite measure of death, any ischemic event, hemorrhagic event, or dementia per patient registry after 3 and 5 years, respectively, from the end of the trial. These outcomes will be published separately.

### Functional MRI exploratory sub-study

#### Calibrated functional MRI

We perform dual-echo pseudo-continuous arterial spin labeling (pCASL) to measure cerebral blood flow (CBF) and blood oxygen level-dependent (BOLD) signals during interleaved normocapnia and hypercapnia (5% CO_2_, 20% O_2_ in N_2_) with/without visual contrast-reversing checkerboard stimulation [28]. From this, we estimate baseline cerebral blood flow (if possible, gray/white-matter perfusion), CVR using hypercapnia, stimulation-induced increase in cerebral metabolic rate of oxygen (CMRO_2_) and perfusion, and the neurovascular coupling index (NVC) defined as the ratio of those two [29].

#### BOLD functional MRI (fMRI)

We assess changes in the regional event-related BOLD response during a peripheral sensory stimulation of the dominant index finger.

#### Diffusion-prepared pCASL (DP-pCASL)

We perform a sequence set of DP-pCASL scans to map the water exchange across the blood–brain barrier (BBB) [30–33]. The outcome is a global brain water coefficient (*k*_*w*_).

See Supplementary material 1 for MRI acquisition and stimulation details (Table 3).

**Table 3:** fMRI outcomes

MRI sequence	Outcome	Details
Calibrated fMRI	CBF/perfusion	Baseline perfusion, gray/white-matter perfusion
	CVR	Increase in perfusion and BOLD signal during hypercapnia
	Visual stimulation response	Visual stimulation-induced increases in CMRO_2_ and CBF
	NVC	Ratio of visual stimulation-induced ΔCMRO_2_ and ΔCBF
BOLD fMRI	BOLD response	Changes in BOLD response to a sensory stimulation
DP-pCASL	BBB water exchange	Water exchange across BBB as a rate constant (*k*_*﻿w*_)

### Cognitive exploratory sub-study

The participants undergo a 1.5-h cognitive assessment evaluating the domains of processing speed, verbal and visual working memory, attention, learning and memory, executive functions, and an estimation of premorbid intelligence by the Danish Adult Reading Test (DART). The following paper-and-pencil and Cambridge Neuropsychological Test Automated Battery (CANTAB) tests are done:

*Paper-and-pencil:* symbol digit modalities test (SDMT), trail making tests A and B, fluency (animal, F-A-S), and digit span forward, backward, ordering, and letter number sequence (from WAIS-IV).

*CANTAB:* motor screening (MOT), spatial working memory (SMW), paired associates learning task (PAL), reaction time (RTI), one-touch stockings of Cambridge (OTS), and rapid visual information processing (RVP).

### Biomarker exploratory sub-study

Blood samples are centrifuged and stored as plasma, serum, and full blood at − 80 °C until the last patient, last visit. Multiplex enzyme-linked immunosorbent assay (Mesoscale, MD, USA) will be used to assess; vascular cell adhesion molecule (VCAM-1), intercellular adhesion molecule-1 (ICAM-1), interleukin-6 (IL-6), tumor necrosis factor alpha (TNF-α), interleukin 1beta (IL-1β), E-selectin, vascular endothelial growth factor, and specific micro-RNAs [20].

### Participant timeline {13}

**Fig. 1:**
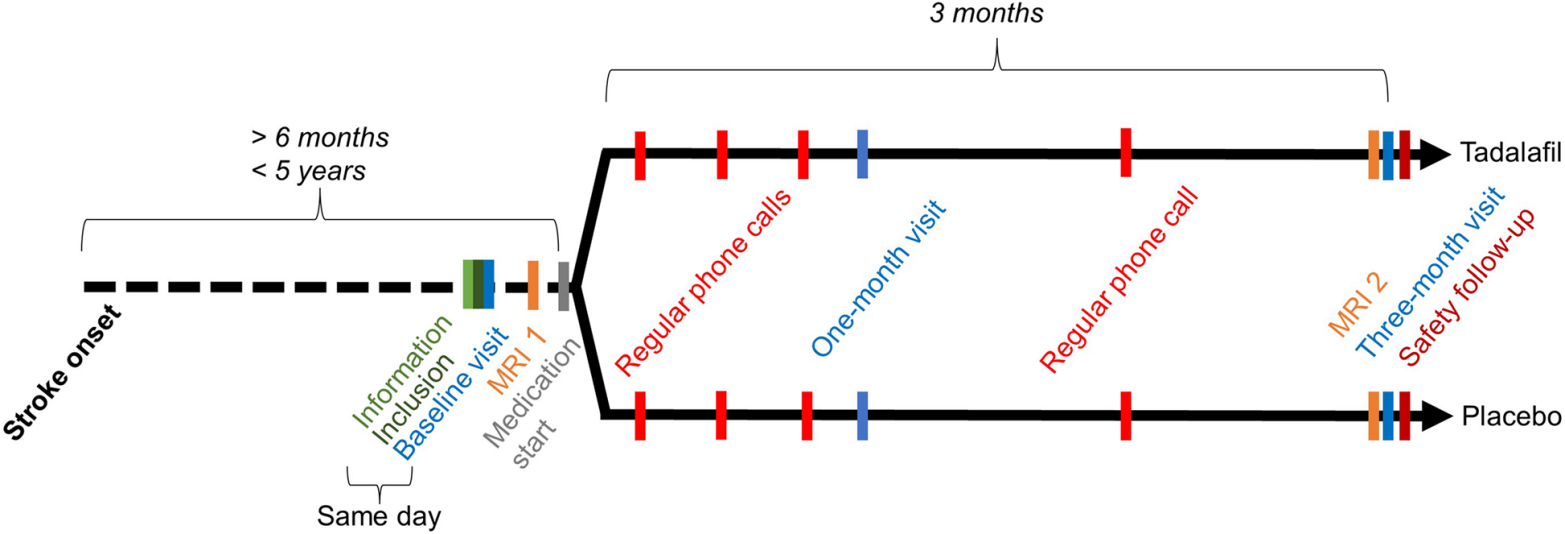
Trial flow chart

Participants complete five on-site study visits. Information, inclusion, and the baseline visit can be conducted on the same day. The second visit includes the first MRI examination of the brain (MRI-1), after which the participants can start the study medication. After 1 month of study medication, a follow-up visit is done to assess the side effects. After 3 months of study medication, the second MRI examination (MRI-2) is conducted followed by the 3-month visit after which the study medication is stopped. The participants are contacted by telephone once weekly for the first 3 weeks after the study medication is initiated and after 2 months to assess the side effects. A safety follow-up telephone visit is performed within 2 weeks after the end of the study medication. Visits are performed at HGH and MRIs are done at DRCMR, AHH. See Fig. 1 and Table 4 for visit details.

**Table 4:** Schedule and timing of standard protocol assessments

Study procedures	Information and inclusion	Baseline visit	MRI-1	Regular phone calls	One-month visit	MRI-2	Three-month visit	Safety follow-up
Information	X							
Informed consent	X							
Medical history		X						
Concomitant medication		X			X		X	
Demographics		X						
Modified Rankin Score (mRS)		X					X	
National Institute of Health Stroke Scale (NIHSS)		X					X	
Cognitive testing		X					X	
MRI			X			X		
Dispensing study medication		X			X			
Study medication tablet count					X		X	
Blood pressure		X			X		X	
Electrocardiogram		X			X		X	
Blood samples: same-day analysis		X					X	
Blood samples: biomarkers		X					X	
Adverse events assessment		X	X	X	X	X	X	X

### Sample size {14}

We hypothesize that 90% of patients in both arms will reach the target dose and complete the trial. By non-inferiority test with a power of 80% and significance level of 0.05, we can detect a difference of 20% between those reaching the target dose on tadalafil compared to placebo with a total sample size of 64 patients. A difference of 20% compared to placebo and 30% in absolute percentage not being able to comply with treatment is considered relevant if to be used in clinical settings. Sample size calculation was also performed for CBF based on 3 T arterial spin labeling (ASL)-MRI for healthy individuals since few 3 T ASL data in patients with CSVD existed when the study was designed. A baseline perfusion of 25 (± 5) ml/100 g/min (mean ± SD) in subcortical white matter, 55 (± 10) ml/100 g/min in cortical gray matter, and 45 (± 10) ml/100 g/min in deep gray nuclei is used for sample size calculations [34–39]. To detect a treatment effect of 15% with a statistical power of 80%, a total sample size of 58 is required in subcortical white matter, 50 for cortical gray matter, and 72 for deep gray nuclei. Based on previous studies, we expect a 15% dropout and 10% of patients are estimated not to reach the target dose. To account for a drop-out of up to 25%, we aim to include 100 patients in total. The sample size is calculated using R software (version 3.6.0).

### Recruitment {15}

Patients are identified by screening hospital records of previously admitted patients in the neurological departments (HGH, BFH, RH, and NZH). Invitation to participate and trial information is sent to the electronic post-box of eligible patients. The written invitation is followed by a phone call after 1–2 weeks to identify interest in participation. Interested patients are invited to the information and inclusion visit.

## Assignment of interventions: allocation

### Sequence generation {16a}

Patients are randomized consecutively to tadalafil or placebo treatment. Randomization procedure and packing of medication are performed by the Capital Region Pharmacy, Copenhagen, Denmark. Randomization is performed 1:1 in variable block sizes. The possibility of block size variation is known to the investigators who enroll participants. However, the blocks are in random order and that order is concealed from the investigators. During inclusion, each participant receives a consecutive randomization number, and the participant is handed out study medication with a matching randomization number.

### Concealment mechanism {16b}

Tadalafil or placebo are packed in 96 opaque capsules in identical appearing containers to conceal which medication the participant has been randomized to. The investigator holds a set of sealed envelopes for each participant where randomization is stated. The envelopes are kept secured and unavailable to study personnel.

### Implementation {16c}

The allocation sequence is performed by the Capital Region Pharmacy, Copenhagen, Denmark. The investigators who enroll participants and conduct intervention are not involved in the randomization procedure.

## Assignment of interventions: blinding

### Who will be blinded {17a}

The trial participants, care providers, and the research team are blinded throughout the whole study from the first patient to the last patient, last visit. Unblinding will be performed after all the trial participants have completed the trial and analyses are done.

### Procedure for unblinding if needed {17b}

In case of adverse events (AE), the trial participants are instructed to contact the study group/investigator. In case of a serious adverse event (SAE), the investigator will assess whether the SAE is related to the study drug as soon as the person becomes aware of the SAE. In this case, the investigator can unblind immediately if necessary. Unblinding can be done by breaking the sealed randomization envelopes.

## Data collection and management

### Plans for assessment and collection of outcomes {18a}

Assessment and collection of outcomes are performed by the study group at the different visits. The medical history of each participant can be collected from the patient’s electronic medical file. Every study delegate who collects data is trained by a qualified trainer for each outcome to ensure the quality of outcomes. We use the following study instruments: MRI scans, neurological examination, questionnaires, cognitive tests performed as paper–pencil tests and computer-based tests, blood samples, blood pressure, electrocardiogram (ECG), and study medication tablet count. A summary of all outcomes is described in the outcomes section.

### Plans to promote participant retention and complete follow-up {18b}

Participant retention is promoted by having close contact with each participant through weekly phone calls for the first months of the study period. Participants are handed out contact information to the study group and are informed that they can contact us at all times in case of questions and emergencies. Participants are also educated about the importance of completing all the visits to be able to assess if the study intervention has any effect on the measured outcomes. If participants discontinue the study, they are asked if we may access their medical records for the next 5 years as a registered-based follow-up to check if the patients have had cerebrovascular events or developed dementia. A participant is not considered lost to follow-up until multiple attempts have been made trying to contact the participant. Each attempt is registered in the electronic case report form (eCRF).

### Data management {19}

All data recorded are kept in the eCRF, REDCap (Vanderbilt University, USA) including medical history, concomitant medication, paper–pencil cognitive tests, questionnaires, adverse events, and blood samples. CANTAB cognitive tests are stored in a logged secure server at the Capital Region of Denmark and MRI data is stored locally at a secure server at DRCMR, AHH. Paper–pencil cognitive tests and questionnaires are performed physically and are therefore also stored in paper format in a physical case report form (CRF). The in-house study visits are also documented in the patients’ electronic medical record except for MRIs which are documented locally in a logbook at DRCMR, AHH. All data are checked for entry and range errors in REDCap after each visit and MRI data are visually checked for quality and artifacts directly after each MRI sequence is performed. After full completion of each subject, the primary investigator signs all eCRFs confirming that they are complete and accurate.

### Confidentiality {27}

Personal information about each participant is stored in REDCap where only authorized study personnel have access. The physical CRFs and the trial master file (TMF) are stored behind two looks to protect against unauthorized access. When exporting data from REDCap to the statistical software, personal identifiers will be removed and data will be pseudo-anonymized. Re-identification can be carried out by the investigator if needed. After the end of the trial and final analysis, data will be fully anonymized.

### Plans for collection, laboratory evaluation, and storage of biological specimens for genetic or molecular analysis in this trial/future use {33}

After the collection of blood samples, they are centrifuged and immediately stored in a local freezer at − 80° at the HGH site behind two locks. After analysis of blood samples, they will be stored in a biobank (BIOSEK) at the HGH site. No genetic analyses are planned to be carried out.

## Statistical methods

### Statistical methods for primary and secondary outcomes {20a}

All outcomes are assessed twice, at baseline, and after 3 months, comparing the two treatment arms. Data will be analyzed for both the intention-to-treat population and the treatment group in the main study. In sub-studies, only the treatment group will be analyzed. The primary outcome will be evaluated by comparing participants achieving target dose by pharmacy count, using a binary logistic regression model with and without adjusting for age, sex, blood pressure, and CSVD score according to STRIVE criteria. MRI outcomes will be analyzed using ANCOVA general linear regression model. MRI data will be adjusted for different factors, such as age, sex, blood pressure, and hematocrit. Cognitive outcomes will likewise be compared using ANCOVA test. All tests, except for the main study outcomes, will be treated as hypothesis-generating, and therefore no multiple comparison correction will be done. A two-sided *P*-value of 0.05 is considered statistically significant. Data is planned to be analyzed using *R* (version 4.3), Microsoft Excel, and REDCap.

### Interim analyses {21b}

An interim analysis will not be done.

### Methods for additional analyses (e.g., subgroup analyses) {20b}

No additional analyses are planned to be performed that are not described in Sect. 20a.

### Methods in analysis to handle protocol non-adherence and any statistical methods to handle missing data {20c}

Data from participants who discontinue the trial before the last visit will be used when possible. Outcomes that are not assessed due to the patient’s withdrawal from the study will be noted as missing data. Missing data will not be imputed.

### Plans to give access to the full protocol, participant-level data, and statistical code {31c}

Fully anonymized data and statistical code will be made available after the trial in public databases.

## Oversight and monitoring

### Composition of the coordinating center and trial steering committee {5d}

The trial is a single-center trial based at the HGH site with multiple collaboration partners. The steering committee includes the primary investigator (PI)/sponsor (CK), the leader at DRCMR, AHH (HRS), MRI post-doc (KNM), professor in psychology (BF), and sub-investigator (SI) and PhD-student (JÖ). All parts of the steering committee provide organizational support, monitor progress, discuss issues, and provide input to the trial. CK and JÖ are responsible for the practical part of running the trial including participant recruiting, data gathering, and analysis of data. HRS, KM, and JÖ are responsible for performing the MRI scans and analyzing MRI data, and BF and JÖ are responsible for collecting and analyzing cognitive data. Quarterly meetings are performed within the steering committee. Weekly meetings are held between the PI and SI. There is no Stakeholder and Public Involvement Group in this trial.

### Composition of the data monitoring committee, its role, and reporting structure {21a}

This trial is monitored by the Good Clinical Practice (GCP) unit in the Capital Region of Denmark. The GCP unit is independent from the sponsor and has no competing interests. The monitor from the GCP unit monitors every aspect of the trial, including participant inclusion, data gathering, and safety of the trial. Frequency of monitor visits depends on enrollment rate and are held approximately for every ten subjects recruited. A written report is generated after each monitor visit describing which participants that have been monitored and findings from the monitoring including data results, adverse events, and safety.

### Adverse event reporting and harms {22}

In case of events and side effects, the trial participants will be instructed to contact the study group/investigator. All side effects and adverse events (AE) during the trial will be recorded in the eCRF and assessed by the PI/SI for causality with the study drug. The Danish summary of product characteristics for Tadalafil Stada is used as a reference document for the assessment of events. All SAEs will be reported to the sponsor within 24 h and once yearly to the Ethics Committee in the Capital Region of Denmark and the Danish Medicines Agency. Suspected unexpected serious adverse reactions (SUSAR) that are life-threatening or fatal will be reported to the Danish Medicines Agency within 7 days. Other suspected unexpected serious adverse reactions that are not life-threatening or fatal will be reported within 15 days. In case of SAEs, the investigator may unblind immediately if necessary.

### Frequency and plans for auditing trial conduct {23}

Direct access to the original data and patient details upon monitoring, auditing, or inspection will be made accessible to the legal authorities (the National/Regional Ethics Committee, The Danish Medicines Agency, The Danish Data Protection Agency, and the GCP-unit). The process can be conducted independently from the investigators and sponsor.

### Plans for communicating important protocol amendments to relevant parties (e.g., trial participants, ethical committees) {25}

Any changes in study design and activities will require a protocol amendment. All amendments must be approved by the Ethics Committee in the Capital Region of Denmark and the Danish Medicines Agency before implementation. Trial registries will also be updated with the amendment.

### Dissemination plans {31a}

Results will be published in peer-reviewed international scientific journals according to the CONSORT guidelines. Results will also be presented at relevant national and international conferences. Trial registers will be updated when there is new information in the trial. All participants will receive a lay summary with the main findings from the trial once the trial is completed and the results have been analyzed. Further lay summary of findings will be provided to patient organizations.

## Discussion

In this randomized, placebo-controlled, double-blind parallel-arm phase 2 clinical trial, we assess the feasibility of daily treatment with tadalafil 20 mg or placebo for 3 months in CSVD patients. Further, we assess tadalafil’s impact on functional cerebrovascular MRI markers of CSVD and cognitive outcomes.

Tadalafil was chosen over other PDE-5 inhibitors since it has the longest plasma half-time of the available PDE5i and due to its documented brain penetrance in rodents [40]. Further, it has been tested in two single-dosing cross-over trials in CSVD patients with good tolerability [20, 24]. We previously found that a single dose of tadalafil increased cortical blood oxygenation measured with near-infrared spectroscopy in CSVD patients, indicating that tadalafil improves cortical perfusion [20]. However, the PASTIS trial found no effect of a single dose of tadalafil on CBF using pCASL-MRI [24]. In the LACI-2 trial, the NO donor isosorbide mononitrate and the PDE-3 inhibitor cilostazol were tested in CSVD patients. They found that treatment was feasible for 1 year with both drugs. Isosorbide mononitrate reduced recurrent stroke risk and cognitive decline. Cilostazol reduced dependence and a combination of both drugs reduced the composite outcome defined as recurrent stroke, cognitive impairment, functional impairment, and death [41]. Hence, this trial showed that it was possible to reduce stroke risk and cognitive decline in CSVD patients by targeting the NO-cGMP pathway.

Feasibility of treatment was selected as the primary outcome since it is essential when repurposing known medication. The MRI protocol was designed to examine key pathological features of CSVD, including the STRIVE criteria [25]. The calibrated fMRI measurements were chosen since it is a non-invasive method to assess CBF, CVR, and NVC [42]. Consequently, these calibrated fMRI measurements may provide proof of concept that tadalafil improves CBF and vessel reactivity. A previous study showed improved event-related BOLD response after sildenafil treatment in patients with Becker Muscular Dystrophy which indicates that PDE-5 inhibitors might only improve on-demand perfusion and not baseline perfusion [43]. NVC addresses changes in local perfusion due to changes in neuronal activity and is therefore an important outcome of this trial [44]. Cerebral BOLD fMRI with sensory stimulation was chosen since it is another method to assess changes in perfusion due to neuronal activity. DP-pCASL is a novel MRI method to address BBB permeability that does not require gadolinium. This outcome was chosen since it is generally accepted that CSVD patients have impaired BBB integrity [45]. The cognitive testing battery was specifically designed to target domains that can be impaired in CSVD patients, e.g., processing speed, verbal and visual working memory, attention, learning and memory, and executive functions [46].

## Trial status

The current protocol version is number 6 dated to 17 May 2022. The first patient was included on 14 June 2022, and on the date of submission of this manuscript (3 April 2024), 64 patients have been included. The trial is estimated to end in late 2024. Three serious adverse events have been reported so far, however, none are adjudicated as caused by trial medication or methods.

### Supplementary Information

Supplementary Material 1.

Supplementary Material 2.

## Data Availability

The investigators will have access to an anonymized final trial dataset containing study results which will be made available in public databases permitted by Danish Law and upon reasonable request directly from the investigators. MRI data can be made available upon reasonable request. Data from medical files and participants are confidential and will remain unavailable.

## Abbreviations

AE: Adverse events
AHH: Amager Hvidovre Hospital
ANCOVA: Analysis of covariance
ASL: Arterial spin labeling
BBB: Blood–brain barrier
BDI-II: Becks Depression Inventory II
BOLD: Blood oxygen level-dependent
CANTAB: Cambridge Neuropsychological Test Automated Battery
CBF: Cerebral blood flow
cGMP: Cyclic guanosine monophosphate
CMRO_2_: Cerebral metabolic rate of oxygen
CRF: Case report form
CSVD: Cerebral small vessel disease
CVR: Cerebrovascular reactivity
DART: Danish Adult Reading Test
DP-pCASL: Diffusion prepared pseudo-continuous arterial spin labeling
DRCMR: Danish Research Centre for Magnetic Resonance
DWI: Diffusion-weighted imaging
ECG: Electrocardiogram
eCRF: Electronic case report form
FLAIR: Fluid-attenuated inversion recovery
fMRI: Functional magnetic resonance imaging
FSS: Fatigue severity scale
GCP: Good clinical practice
HGH: Herlev Gentofte Hospital
ICAM-1: Intercellular adhesion molecule-1
IL-1β: Interleukin 1beta
IL-6: Interleukin-6
IQCODE: Informant Questionnaire on Cognitive Decline in the Elderly
MoCA: Montreal cognitive assessment
MOT: Motor screening
MRI: Magnetic resonance imaging
NO: Nitric oxide
NVC: Neurovascular coupling-index
NZH: North Zealand Hospital
OTS: One-touch stockings of Cambridge
PAL: Paired associates learning task
pCASL: Pseudo-continuous arterial spin labeling
PDE-5: Phosphodiesterase 5
PI: Primary investigator
RH: Rigshospitalet
RTI: Reaction time
RVT: Rapid visual information processing
SAE: Serious adverse event
SDMT: Symbol digit modalities test
SMW: Spatial working memory
STRIVE: STandards for ReportIng Vascular changes on nEuroimaging
SUSAR: Suspected unexpected serious adverse reaction
SWI: Susceptibility weighted imaging
TIA: Transitory ischemic attack
TMF: Trial master file
TNF-α: Tumor necrosis factor alpha
TOF: Time of flight
VCAM-1: Vascular cell adhesion molecule 1
WHO-5: World Health Organization 5
WMH: White matter hyperintensity
