# Supplementary material for: Cerebral blood flow and cognition after 3 months tadalafil treatment in small vessel disease (ETLAS-2): study protocol for a randomized controlled trial
Source: Trials. 2024 Aug 29;25:570. doi: 10.1186/s13063-024-08402-4 (PMC11360322; doi:10.1186/s13063-024-08402-4)
Supplement: Supplementary file 1 — Supplementary Material 1. [file 13063_2024_8402_MOESM1_ESM.docx]

| Sequence | T1w | T2w | FLAIR | SWI | DWI | TOF | Dual echo pCASL | DP-pCASL | fMRI BOLD |
| --- | --- | --- | --- | --- | --- | --- | --- | --- | --- |
| Voxel size | 0.85 x 0.85 x 0.85 | 0.85 x 0.85 x 0.85 | 1.0 x 1.0 x 1.0 | 0.60 x 0.60 x 1.5 | 1.51 x 1.89 x 4.00 | 1.50 x 1.50 x 1.50 | 3.75 x 3.90 x 7.00 | 3.81 x 3.77 x 7.00 | 3.00 x 3.00 x 3.00 |
| FoV (mm) | 245 x 245 x 208 | 245 x 245 x 190 | 256 x 256 x 202 | 230 x 189 x 160 | 230 x 230 x 159 | 150 x 78 x 156 | 240 x 240 | 238 x 238 | 192 x 192 x 126 |
| TR (ms) | shortest (6.0) | 2500 | 4800 | 31 | shortest (3856) | shortest (25) | 4500 | 5700/5900/4500 | 2500 |
| TE (ms) | shortest (2.7) | shortest (270) | shortest (331) | 4 echoes, first echo: 7.2 ms, delta TE: 6.2 ms | shortest (84) | shortest (2.3) | 11 & 30 | 18 | 30 |
| Flip angle (°) | 8 | 90 | 40 | 17 | 90 | 16 | 90 | 90 | 80 |
| Acquisition time (min:sec) | 05:41 | 06:33 | 05:41 | 03:43 | 00:46 | 03:06 | 24:09 | 22:33 | 05:08 |
| Readout method (TFE/TSE factor) | 3D TFE (243) | 3D TSE (133) | 3D TSE (182) | 3D FFE | 2D SE-EPI | 3D FFE | FEE/EPI | FEE/EPI | 2D FFE/EPI |
| SENSE/halfscan factor | SENSE 2.0 (AP) | SENSE 2.0 (AP), 1.8 (RL) | SENSE 1.8 (AP), 1.9 (RL) | SENSE 2.0 (RL), 1.29 (FH) | SENSE 2.0 (AP)/halfscan factor 0.841 | n.a. | SENSE 2.0 (AP) |  | n.a. |
| Other |  |  | Inversion time 1650 ms |  |  |  | *1 | *2 | *3 |

Supplementary material 1: Table over MRI details for the applied sequences.

*1: Dual echo pCASL:

FOV: 240 x 240 mm, mat: 64x61, slices: 14, thickness: 7mm, EPI factor: 63, labeling duration: 1650ms, post-labeling duration: 2000ms, global background suppression: 1680ms & 3150 ms, TE:11 & 30 ms, TR: 4500, 160 dynamics, label/control interleaved.

M0-scan: same, but no background suppression or labeling, TR: 6000 ms

Visual stimulation: Contrast-reversing checkerboard at temporal flicker frequency of 8 Hz for 36 seconds.

Monitoring: Heat rate, peripheral oxygen saturation, respiration frequency, expiratory CO2.

Paradigm:


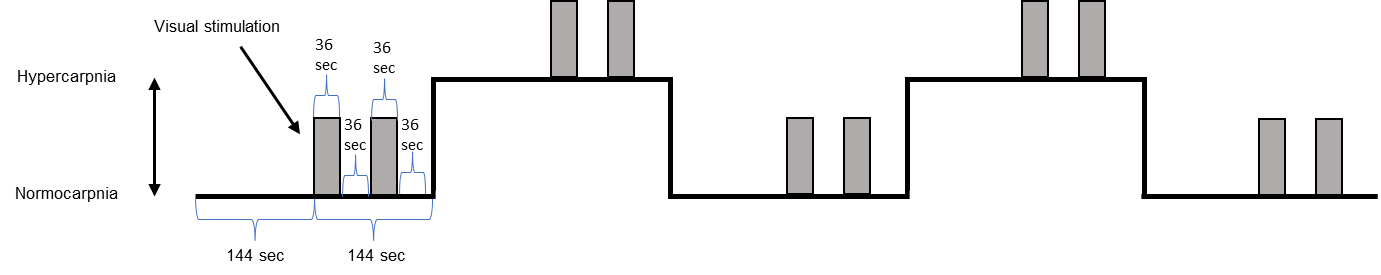


*2: DP-pCASL:

FOV: 238 x 238 mm, mat: 64x63, slices: 14, thickness: 7mm, EPI factor: 63, labeling duration: 1650ms, global background suppression: 1680ms & 3150 ms

b-value: 50 s/mm^2, pld: 2000ms TE: 18ms, TR: 5700ms, 30 dynamics

b-value: 0 s/mm^2, pld: 2000ms TE: 18ms, TR: 5700ms, 30 dynamics

b-value: 14 s/mm^2, pld: 900ms TE: 19ms, TR: 4500ms, 30 dynamics

b-value: 0 s/mm^2, pld: 900ms TE: 19ms, TR: 4500ms, 30 dynamics

M0-scan: same but no background suppression, b-value: 0, TE: 18ms TR: 5900ms, 2 dynamics

*3: fMRI BOLD

Stimulation is performed using Digitimer DS5 stimulator (Digitimer Ltd, UK). Each stimulation is done as 20 pulse cycles with 5 Hz frequency, 200 µs pulse width. Stimulation amplitude is set to three times sensory threshold which is calibrated for each scan.
